# Interface Modulation and Lithium Dendrite Suppression of LLTO via Synergistic KH560-PDA Co-Grafting for PVDF-HFP Composite Solid Electrolytes

**DOI:** 10.3390/ma19143113

**Published:** 2026-07-20

**Authors:** Dingqin Wang, Zihao Fei, Deyi Zheng

**Affiliations:** 1 College of Materials and Metallurgy, Guizhou University, Guiyang 550025, China; wdqqqqq0628@163.com; 2 Guiyang High-Tech YiGe Electronic Co., Ltd., Guiyang 550022, China; 15085424820@163.com

**Keywords:** Li_0.33_La_0.56_TiO_3_, KH560, composite solid electrolyte, interface regulation, lithium dendrite suppression

## Abstract

Poly (vinylidene fluoride-co-hexafluoropropylene) (PVDF-HFP) polymer electrolytes suffer from low ionic strength and poor mechanical performance. Meanwhile, lithium lanthanum titanate (LLTO) fillers exhibit severe agglomeration and weak interfacial compatibility with the polymer matrix. To solve these problems, 3-glycidoxypropyltrimethoxysilane (KH560) at four different concentrations (1 wt%, 2 wt%, 3 wt%, 4 wt%) was loaded onto polydopamine-modified LLTO (PDA@LLTO). The modified materials were mixed with PVDF-HFP, and composite solid electrolytes were fabricated by the solution casting method. The epoxy groups in KH560 undergo ring-opening reactions with amino and hydroxyl moieties on PDA, while its trimethoxysilane groups crosslink with the polymer matrix, forming a robust “LLTO-PDA-KH560-polymer” interfacial structure. This dual modification markedly improves the dispersion of PDA@LLTO, strengthens interfacial adhesion, and enhances the mechanical and electrochemical properties of the composite electrolyte. All KH560 loadings suppress LLTO agglomeration, and the 3 wt% grafting ratio yields the optimal performance: a uniform and dense microstructure, a room-temperature ionic conductivity of 5.92 × 10^−4^ S cm^−1^, an electrochemical stability window extended to 4.88 V, and a tensile strength over 50% higher than the ungrafted sample. The modified electrolyte effectively inhibits lithium dendrite growth and enhances the cycling stability of solid-state batteries. This work demonstrates that KH560-PDA synergistic modification enables comprehensive performance optimization of composite electrolytes, offering a viable strategy for designing high-performance electrolytes for solid-state lithium-metal batteries.

## 1. Introduction

Composite solid electrolytes (CSEs) combine the flexibility and processability of polymer matrices with the high ionic conductivity and mechanical robustness of inorganic fillers, thereby overcoming inherent drawbacks of single-component systems and achieving synergistic improvements in electrochemical performance, interfacial compatibility, and mechanical strength [1,2]. Widely studied polymer matrices include polyethylene oxide (PEO), poly (vinylidene fluoride-co-hexafluoropropylene) (PVDF-HFP), polyacrylonitrile (PAN), and polymethyl methacrylate (PMMA) [3,4]. Representative inorganic fillers comprise garnet-type oxides (LLZO), perovskite-type oxides (LLTO), NASICON-type oxides, and sulfide electrolytes [5]. Rational compositional and interfacial engineering enables the construction of continuous, efficient Li^+^-transport pathways within the electrolyte, which is critical for developing high-safety, high-energy-density solid-state lithium batteries [6].

PVDF-HFP is one of the most prevalent polymer matrices for solid electrolytes owing to its excellent chemical stability, processability, and ability to solvate lithium ions [7]. However, neat PVDF-HFP suffers from low room-temperature ionic conductivity (<10^−6^ S cm^−1^) and insufficient mechanical strength, which restrict its application in high-performance solid-state lithium-metal batteries [8]. To address these issues, the incorporation of inorganic fast-ion conductors such as LLTO has become a mainstream modification strategy. LLTO is particularly attractive due to its high ionic conductivity, good chemical stability, and wide electrochemical stability window. Nonetheless, the high polarity of pristine LLTO leads to a large interfacial energy mismatch with the hydrophobic PVDF-HFP matrix, resulting in poor interfacial compatibility [9]. Moreover, the high surface energy of LLTO particles causes severe agglomeration in the polymer matrix, which not only compromises its Li^+^ transport contribution but also disrupts the electrolyte microstructure and degrades battery cycling stability [10,11]. Therefore, surface modification of LLTO to improve interfacial compatibility with PVDF-HFP, suppress particle agglomeration, and realize synergistic enhancement of overall electrolyte performance has become a central challenge in this field.

**Figure 1:**
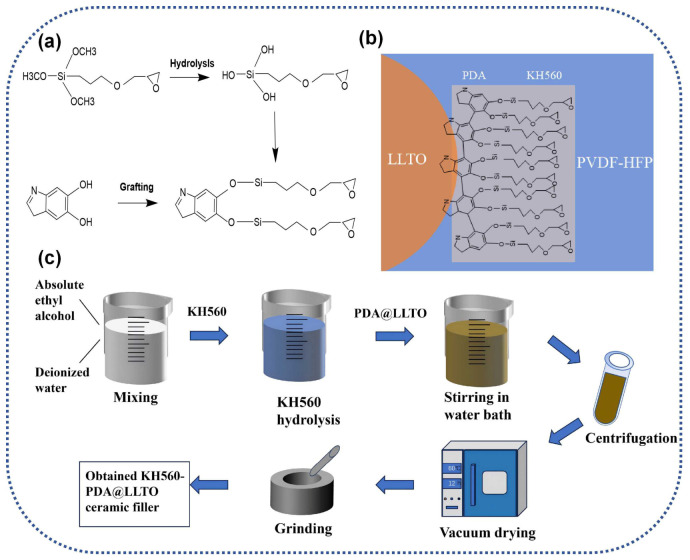
(**a**) Schematic illustration of KH560 hydrolysis and grafting process. (**b**) Schematic of KH560-PDA grafting onto the LLTO surface. (**c**) Preparation flow chart of KH560-PDA@LLTO.

Polydopamine (PDA) can form a uniform coating on LLTO via oxidative self-polymerization, which improves particle dispersion and strengthens interfacial adhesion between LLTO and the polymer matrix [12,13]. Jia, Mengya et al. [14] reported that PDA coating on LLTO enhanced interfacial bonding between LLTO and PVDF, yielding a bilayer composite electrolyte with superior interfacial contact and processability; the corresponding Li/Ni_0.6_Mn_0.2_Co_0.2_ cell delivered 158.2 mAh/g at 0.1 C and retained 83% capacity after 100 cycles. Manuela Ferreira et al. [15] adopted polydopamine (PDA) to boost the interfacial adhesion between inorganic LLZO garnet ceramic and PEO polymer electrolyte. The PDA interfacial modification effectively addresses the poor compatibility between LLZO ceramic and PEO matrix, optimizes the processability of composite slurry, improves the interfacial wettability and intimate contact of two phases, and drastically reduces interfacial impedance. After modification, the room-temperature Li-ion conductivity of the hybrid solid electrolyte reaches the industrial benchmark of 10^−4^ S cm^−1^, showing promising prospects for all-solid-state lithium battery applications. These studies confirm that PDA coating effectively mitigates particle agglomeration and the associated issues of current localization and reduced conductivity. Although PDA coating improves LLTO dispersion, the abundant hydrophilic amino and hydroxyl groups on PDA still cause interfacial repulsion with hydrophobic polymers, leading to internal defects. As a silane coupling agent, KH560 contains an epoxy group that reacts with amino/hydroxyl groups on PDA and a trimethoxysilane group that crosslinks with the polymer matrix [16,17]. The schematic diagram of KH560 hydrolysis is shown in Figure 1a. Yunqing Xia et al. [18] showed that co-modification of g-C_3_N_4_ with PDA and KH560 greatly improved dispersion in aqueous solution and compatibility with waterborne epoxy resin. Chi et al. [19] recently realized interfacial functionalization of three-dimensional porous LLTO frameworks via silane coupling agent KH560. This strategy optimizes the ceramic/polymer interface through covalent bonds, reduces the lithium-ion migration energy barrier and inhibits the reduction of Ti^4+^. The as-prepared solid polymer electrolyte delivers an ionic conductivity of 2.37 S cm^−3^ at 60 °C, and the Li symmetric cell achieves stable Li plating/stripping for 4000 h. The full battery paired with an LFP cathode retains 82.4% of its initial capacity after 1200 cycles at 0.2 C. The above two studies verify that both PDA and KH560 silane coupling agents can effectively enhance the interfacial compatibility between fillers and matrixes, yet they rely on distinct interaction mechanisms to optimize material performance in different composite systems.

Against this background, this work introduces KH560 grafting into the PDA@LLTO/PVDF-HFP system to construct a stable “LLTO-PDA-KH560-polymer” interface, as shown in Figure 1b. This dual modification further improves the dispersion of PDA@LLTO and reinforces filler–matrix interfacial interactions, thereby enhancing mechanical properties and electrochemical stability. By varying the KH560 grafting ratio (1 wt%, 2 wt%, 3 wt%, 4 wt%), we investigated its effects on filler dispersion and interfacial compatibility, fabricated high-performance KH560-PDA@LLTO/PVDF-HFP composite solid electrolytes, and systematically characterized their microstructure, mechanical properties, and electrochemical performance. The optimal KH560 loading is identified, providing experimental and theoretical support for designing advanced composite electrolytes for solid-state lithium-metal batteries.

## 2. Materials and Methods

### 2.1. Materials

PVDF-HFP powder (average Mw ≈ 455,000) was supplied by Aiwei Scientific Research (Shanghai, China). Dopamine hydrochloride, tris (hydroxymethyl) aminomethane (Tris), lithium bis(trifluoromethanesulfonyl) imide (LiTFSI, 99.99%), N, N-dimethylformamide (DMF), 3-glycidoxypropyltrimethoxysilane (KH560), and methanol were purchased from Aladdin (Shanghai, China). Li_0.33_La_0.56_TiO_3_ (LLTO) microparticles were synthesized via a solid-state reaction method.

### 2.2. Experimental Scheme

(1) After grafting PDA@LLTO with KH560, KH560-PDA@LLTO, PDA@LLTO and LLTO were characterized by XRD, FT-IR and XPS to evaluate the grafting effect. (2) The grafted fillers and ungrafted fillers were separately added into the matrix to prepare composite membranes of KH560-PDA@LLTO/PVDF-HFP, PDA@LLTO/PVDF-HFP and LLTO/PVDF-HFP (marked as K-PLP, PLP and LP respectively), which were then tested for XRD, SEM, thermal stability and mechanical properties. (3) Fillers with different grafting rates were prepared and further fabricated into composite membranes for electrochemical performance tests.

### 2.3. Preparation Procedures

(1) Synthesis of KH560-PDA@LLTO: PDA@LLTO was first prepared: 36 mg Tris and dopamine hydrochloride (2.4% of the mass of LLTO) were dissolved in 30 mL methanol, followed by the addition of 2.5 g LLTO powder. The brown suspension was stirred at 500 rpm for 16 h under ambient conditions, then washed 2~3 times with methanol and dried at 80 °C under vacuum for 24 h to obtain PDA@LLTO. For KH560 grafting: PDA@LLTO was dispersed in anhydrous ethanol and ultrasonicated for 20 min. KH560 (1%, 2%, 3%, 4% of PDA@LLTO mass) was added, and the mixture was stirred at 60 °C for 6 h to allow ring-opening reactions between epoxy groups of KH560 and amino groups of PDA. The product was centrifuged, washed 2~3 times with anhydrous ethanol, and vacuum-dried at 80 °C for 8 h to yield KH560-PDA@LLTO. After KH560 grafting, the color of PDA@LLTO particles changed from white to brown.
(2) Fabrication of KH560-PDA@LLTO/PVDF-HFP Membranes: Composite electrolyte films were prepared via solution casting. PVDF-HFP was dissolved in DMF at 60 °C under stirring. LiTFSI and KH560-PDA@LLTO powder were added, ultrasonicated for 30 min, and stirred for 12 h. The homogeneous slurry was degassed in vacuum, cast onto a substrate, and dried at 60 °C under vacuum for 10 h. The preparation process is shown in Figure 1c. The resulting film was punched into 16 mm-diameter disks for coin-cell assembly. The composite films with different KH560 grafting amounts were labeled as 1 wt% K-PLP, 2 wt% K-PLP, 3 wt% K-PLP, and 4 wt% K-PLP, respectively. Secondly, PDA@LLTO/PVDF-HFP is designated as PLP, while LLTO/PVDF-HFP is designated as LP.

### 2.4. Material Characterization

The crystalline phase structures of LLTO samples and composite solid electrolytes (CSE) were characterized by an X-ray diffractometer (XRD, Rigaku Ultima IV, Tokio, Japan); Fourier transform infrared spectroscopy (FTIR, Thermo Scientific Nicolet iS20, Waltham, MA, USA) was used to compare the differences in functional groups among LLTO, PDA@LLTO and KH560-PDA@LLTO to verify the grafting effect of KH560; X-ray photoelectron spectroscopy (XPS, Thermo Fisher-K-Alpha, Waltham, MA, USA) was adopted to analyze the surface elemental composition and chemical states of LLTO and KH560-PDA@LLTO; a scanning electron microscope (SEM, ZEISS Sigma 300, Oberkochen, Germany) was utilized to observe the surface and cross-sectional morphologies of composite solid electrolyte (CSE) membranes, as well as the surface state of lithium sheets after cycling; and the CMT4503 (Haining, Zhejiang, China) universal tensile testing machine was employed for mechanical property tests. Thermogravimetric (TG) analysis of CSE membranes was carried out by a thermogravimetric analyzer (TG, METTLER TOLEDO TGA/DSC1, Greifensee, Switzerland) under a nitrogen atmosphere, with a heating range from 30 °C to 800 °C and a heating rate of 10 °C/min, to evaluate their thermal stability; optical images of the as-prepared samples were captured using a digital camera to directly observe the appearance and morphology of the samples.

### 2.5. Electrochemical Measurements

The ionic conductivity was tested by assembling stainless steel (SS) SS/CSE/SS sandwich-type batteries, with a test frequency ranging from 10^−2^ Hz to 10^5^ Hz. The ionic conductivity is calculated according to the formula σ = L/(RS), where (R) is the measured resistance of the solid composite electrolyte, (L) is the thickness of the solid composite electrolyte membrane, and (S) is the electrode area. The electrochemical stability window was determined by linear sweep voltammetry (LSV) using assembled Li/CSE/SS batteries within a potential range of 2.0–6.0 V at a scan rate of 1.0 mV/s. The lithium-ion transference number was tested by assembling Li/CSE/Li batteries with a polarization voltage of 10 mV. The assembled coin cells were subjected to galvanostatic cycling tests at a current density of 0.1 mA/cm^2^. Charge discharge tests were carried out at room temperature under cycling rates of 0.5 C, 1 C and 2 C, followed by rate performance tests.

### 2.6. Cathode Preparation and Cell Assembly

LiFePO_4_ (LFP), Super P, and PVDF were mixed at a weight ratio of 8:1:1, dispersed in N-methyl-2-pyrrolidone (NMP), coated onto Al foil, and dried at 90 °C under vacuum for 12 h. Cathodes were punched into 12 mm disks. Its load density is 1.63 mg/cm^2^. CR2032 coin cells were assembled in an Ar-filled glove box in the sequence: negative case, Li foil, CSE, cathode, spacer, spring, and positive case. A small amount (5 μL) of liquid electrolyte was added at the interfaces to improve wettability.

## 3. Results and Discussion

### 3.1. Characterization of KH560-Grafted PDA@LLTO

This section evaluates the grafting efficiency of KH560 by comparing the grafted KH560-PDA@LLTO filler, coated PDA@LLTO, and unmodified LLTO using XRD, FT-IR, and XPS testing methods.

**Figure 2:**
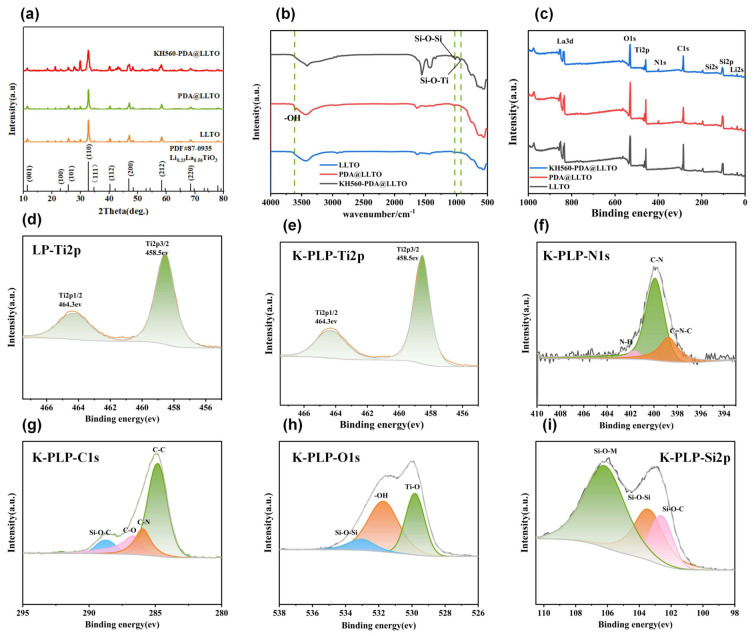
(**a**) XRD patterns of KH560-PDA@LLTO, PDA@LLTO, and LLTO. (**b**) FT-IR spectra of KH560-PDA@LLTO, PDA@LLTO, and LLTO. (**c**) Full XPS spectra of KH560-PDA@LLTO and PDA@LLTO. (**d**,**e**) High-resolution Ti 2p spectra of LLTO and KH560-PDA@LLTO. (**f**) High-resolution N 1s spectrum of KH560-PDA@LLTO. (**g**–**i**) High-resolution C1s, O1s, and Si2p spectra of KH560-PDA@LLTO.

The phase structure is a core factor determining the intrinsic properties of inorganic fillers and composite electrolytes. In this study, X-ray diffraction (XRD) was used to systematically characterize the crystal structures of LLTO inorganic fillers and composite electrolytes. Figure 2a shows the XRD patterns of LLTO, PDA@LLTO, and KH560-PDA@LLTO fillers, with the characteristic peak positions and crystal plane indices of the standard PDF (#87-0935) for Li_0.33_La_0.56_TiO_3_ marked at the bottom. For the inorganic fillers, the diffraction peak positions of the three samples (LLTO, PDA@LLTO, and KH560-PDA@LLTO) are completely consistent with the standard card, indicating that the prepared LLTO-based fillers all possess a pure-phase perovskite crystal structure [20].

To verify the success of PDA coating and KH560 grafting modification, FTIR characterization was performed on PDA@LLTO and KH560-PDA@LLTO samples, and the results are shown in Figure 2b. In the spectrum of the PDA@LLTO sample, the absorption peaks of the characteristic functional groups of PDA molecules can be observed, confirming that PDA has been successfully coated on the surface of LLTO. The KH560-PDA@LLTO sample exhibits new characteristic absorption peaks at approximately 1100 cm^−1^ and 950 cm^−1^, which are attributed to the stretching vibration of Si-O-Si and the characteristic peak of Si-O-Ti, respectively, indicating that KH560 has been successfully grafted onto the surface of PDA@LLTO particles. Compared with PDA@LLTO, the -OH stretching vibration peak around 3400 cm^−1^ disappears for KH560-PDA@LLTO, which is due to the hydrogen bonding formed between the silanol groups of KH560 and the hydroxyl groups on the surface of PDA@LLTO [21]. The above results demonstrate that KH560 successfully achieves the surface silanization modification of LLTO particles by reacting with the hydroxyl groups on the surface of PDA@LLTO, introducing silicon-containing functional groups on its surface and improving the interfacial compatibility between inorganic fillers and organic polymer matrices [22].

To further accurately analyze the surface elemental composition, chemical valence states and chemical bonding modes of modified LLTO particles, XPS survey spectra and high-resolution spectra characterizations were performed on PDA@LLTO and KH560-PDA@LLTO samples. The survey spectrum results showed that compared with LLTO, PDA@LLTO exhibited the C1s peak attributed to PDA, indicating that PDA was successfully coated on the surface of LLTO as shown in Figure 2c. In comparison with LLTO and PDA@LLTO, KH560-PDA@LLTO displayed characteristic Si2p and Si2s peaks at 103.8 eV and 153.2 eV, while characteristic peaks such as La3d, Ti2p, O1s, C1s and N1s still existed, demonstrating that KH560 was successfully grafted onto the surface of PDA@LLTO. In Figure 2d–i, the peak-differentiation and fitting analysis of high-resolution spectra further revealed the surface chemical bonding environment: after PDA coating and KH560 grafting on PDA@LLTO, the peaks in Ti2p showed no shift and no additional impurity peaks, which further confirmed that coating and grafting would not damage the intrinsic crystal structure and valence state integrity of LLTO. The coexistence of C-N and C=N-C two peaks in the N1s spectrum verified the presence of the PDA layer, confirmed the completion of the dopamine self-polymerization coating process, and indicated that the chemical structure of PDA was not destroyed during the grafting process [23]; the C-C, C-N, C-O and Si-O-C peaks in the C1s spectrum cross-verified the existence of the PDA carbon skeleton, KH560 alkyl chains and interfacial bonding structures; the Ti-O, -OH, and Si-O-Si peaks in the O1s spectrum corresponded to LLTO lattice oxygen, PDA phenolic hydroxyl groups and KH560 siloxane networks respectively, further proving the construction of the composite modification layer; and the fitted Si-O-C and Si-O-Si bonds in the Si2p spectrum were derived from KH560 molecules, while the formation of Si-O-M originated from the reaction between Si-OH and hydroxyl groups on the surfaces of PDA and LLTO, which further confirmed that KH560 was grafted onto the surface of PDA@LLTO through hydrolysis and condensation reactions [24,25]. In summary, the KH560-PDA coating completely covers the particle surface and finally forms KH560-PDA@LLTO composite powder with a stable structure and excellent interfacial bonding.

### 3.2. Microstructure and Physical Properties of Composite Membranes

This section compares the XRD, SEM, dispersion properties, and mechanical properties of three composite membranes: KH560-PDA@LLTO/PVDF-HFP, PDA@LLTO/PVDF-HFP, and LLTO/PVDF-HFP.

**Figure 3:**
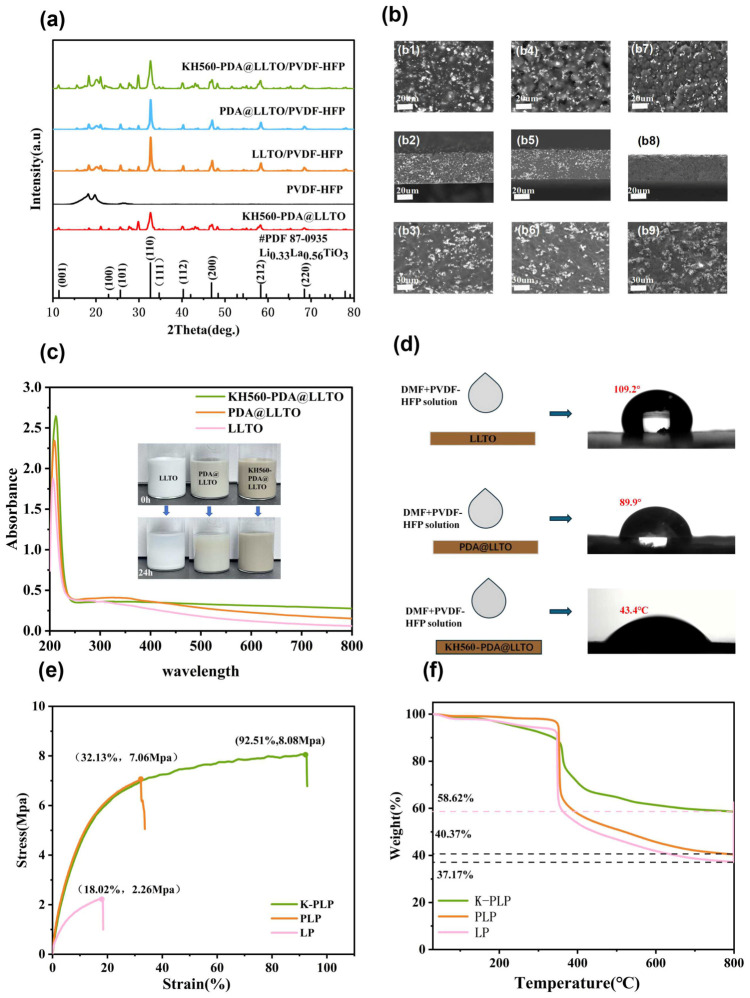
(**a**) XRD patterns, (**b**) SEM of composite solid electrolytes with various fillers: (**b1**,**b4**,**b7**) Surface morphologies of LP, PLP, K-PLP membranes; (**b2**,**b5**,**b8**) Cross-section views of corresponding films; (**b3**,**b6**,**b9**) Magnified cross-sectional microstructures. (**c**) UV–Vis absorption spectra, (**d**) contact angle curves, (**e**) mechanical performance plots and (**f**) thermal stability profiles of LP, PLP and K-PLP composite electrolytes.

As shown in Figure 3a, in the composite electrolyte system, pure PVDF-HFP exhibits typical characteristic diffraction peaks of the polymer at 2θ ≈ 18° and 20°, corresponding to its crystalline phase structure; in the pattern of the KH560-PDA@LLTO/PVDF-HFP composite electrolyte, both the polymer characteristic peaks of PVDF-HFP and the perovskite characteristic peaks of KH560-PDA@LLTO are retained simultaneously. There is no obvious shift in the positions of the two types of characteristic peaks, and no new impurity phase diffraction peaks appear. This result fully proves that during the preparation of the composite electrolyte, only physical blending occurs between the KH560-PDA@LLTO inorganic filler and the PVDF-HFP polymer matrix, without chemical reactions taking place. The inorganic filler maintains favorable phase stability in the polymer matrix, laying a structural foundation for the composite electrolyte to combine the flexibility of polymers and the high ion transport performance of inorganic fillers.

The microscopic morphology of composite electrolytes and the dispersion of inorganic fillers directly affect their ion transport efficiency and mechanical properties [26]. Figure 3b presents the surface, low-magnification cross-section and high-magnification cross-section SEM images of LP, PLP and K-PLP composite electrolytes. The surface images (b1, b4, b7) reveal severe filler agglomeration in pristine LP, slightly alleviated aggregation in PLP with single PDA coating, while uniformly dispersed LLTO particles without obvious agglomerates are observed in K-PLP modified by KH560/PDA co-grafting, owing to improved interfacial compatibility between inorganic fillers and PVDF-HFP matrix. Low-magnification fracture cross-sections (b2, b5, b8) demonstrate that LP and PLP membranes possess abundant internal continuous loose structures, whereas K-PLP exhibits a continuous and compact cross-section with drastically reduced structural defects. High-resolution cross-section images (b3, b6, b9) further confirm that aggregated LLTO particles exist in LP and PLP, while evenly distributed fillers in K-PLP construct continuous lithium-ion transport pathways. Collectively, the synergistic KH560/PDA co-grafting strategy effectively homogenizes LLTO dispersal of the polymer and optimizes the compact microstructure of polymer electrolyte, which contributes to enhanced ionic conductivity and interfacial stability.

The results of UV-Vis absorption spectroscopy and contact angle measurements jointly confirm that KH560 grafting modification achieves dual optimization of the dispersibility and interfacial compatibility of PDA@LLTO fillers. In the spectrum of Figure 3c, KH560-PDA@LLTO exhibits significantly higher absorbance than PDA@LLTO in the full wavelength range of 200~800 nm. This is attributed to the strong interaction formed between the epoxy groups on the surface of KH560 and solvent molecules, which effectively inhibits the agglomeration of LLTO particles, enables the fillers to disperse in a more uniform state, reduces the light scattering loss caused by agglomerates, and lays a foundation for constructing a continuous filler distribution within the composite membrane [27]. The contact angle between LLTO and the PVDF-HFP polymer solution reaches up to 109.2° in Figure 3d. The contact angle test results verify the improvement of interfacial the PVDFerties: the contact angle between PDA@LLTO and PVDF-HFP polymer solution is 89.9°, showing weak wettability and poor interfacial compatibility; after KH560 grafting, the Si-O bonds and epoxy groups introduced on the filler surface significantly enhance the polarity, causing the contact angle to drop sharply to 43.4° and greatly improving the wettability between inorganic fillers and the organic polymer matrix. Such improved interfacial compatibility not only strengthens the bonding strength between fillers and the matrix, but also effectively reduces the interfacial impedance, removing obstacles for the rapid transport of lithium ions at the filler–polymer interface, and ultimately synergistically promoting the enhancement of ionic conductivity and lithium ion transference number of composite solid electrolytes.

The mechanical properties of solid electrolytes are one of the key indicators for their application in solid-state lithium batteries. Excellent mechanical strength can effectively inhibit the penetration of lithium dendrites, while appropriate toughness can meet the deformation requirements during battery assembly [28,29]. In this study, the stress–strain curves of PLP and K-PLP composite electrolytes were tested using a universal testing machine, and the results are shown in Figure 3e. LP exhibits a fracture strength of 2.26 MPa and an elongation at break of only 18.02%, presenting a mechanical characteristic of “exhibits high strength but low toughness”, which is a typical mechanical behavior of pure PVDF-HFP polymer matrix and makes it difficult to withstand the volume change of lithium-metal anodes during battery cycling. The PLP sample has a fracture strength of 7.06 MPa and an elongation at break of 32.13%. For the K-PLP sample incorporated with KH560-PDA@LLTO, the mechanical properties are significantly optimized: its fracture strength increases to 8.08 MPa, which is a 14.40% improvement compared with PLP; the elongation at break rises sharply to 92.51%, 2.88 times that of PLP, achieving simultaneous enhancement of strength and toughness. The improvement mechanism of mechanical properties mainly stems from two aspects: first, strong interfacial bonding is formed between the KH560-PDA@LLTO inorganic filler and the PVDF-HFP matrix through Si-O-C covalent bonds and intermolecular forces. When the electrolyte is subjected to external forces, the filler can effectively bear loads and hinder the sliding of polymer chain segments, thereby improving mechanical strength; second, the flexible chain segments of PDA and the organic groups of KH560 act as “plasticizers”, improving the chain segment mobility of the polymer matrix and thus significantly enhancing toughness [30]. The optimized mechanical properties enable the K-PLP composite electrolyte to not only effectively resist lithium dendrite penetration, but also adapt to the deformation demands of battery assembly and cycling, greatly improving the safety of solid-state batteries.

Thermal stability is a core indicator for evaluating the safety performance of solid electrolytes [31]. In this study, thermogravimetric (TG) analysis was adopted to assess the thermal decomposition behaviors of LP, PLP and K-PLP composite electrolytes, and the results are presented in Figure 3f. The thermogravimetric curves of the three samples all exhibit a typical “single-stage weight loss” characteristic, with the main weight loss range concentrated at 350~400 °C. This weight loss process corresponds to the thermal decomposition and defluorination reaction of the PVDF-HFP polymer matrix. A comparison of the thermal decomposition behaviors of different samples shows that the initial thermal decomposition temperature of pure LP is approximately 350 °C, and the residual mass at 800 °C is 62.83%. The PLP sample has an initial thermal decomposition temperature of about 350 °C and a residual mass of 59.63% at 800 °C; after introducing KH560-PDA@LLTO, the initial thermal decomposition temperature remains at 350 °C, while the residual mass at 800 °C is only 41.38%. This indicates that the introduction of inorganic fillers modified by KH560-grafted PDA coating effectively inhibits the thermal motion of polymer chain segments. The core reason for the improved thermal stability lies in the following aspects: the KH560-PDA@LLTO inorganic fillers form a continuous heat-resistant skeleton in the polymer matrix, which can support the polymer structure at high temperatures and delay the escape of thermal decomposition gases [32]; meanwhile, the strong interfacial interaction between the fillers and the polymer matrix restricts the thermal vibration and sliding of PVDF-HFP molecular chains, and increases the thermal decomposition activation energy of the polymer matrix [33].

### 3.3. Electrochemical Performance

This section evaluates KH560-PDA@LLTO/PVDF-HFP with varying grafting ratios (1 wt%, 2 wt%, 3 wt%, 4 wt%) through comprehensive electrochemical performance tests to identify the optimal grafting ratio. The study also demonstrates that grafting modification of KH560 effectively inhibits lithium dendrite growth and significantly enhances electrochemical performance.

**Figure 4:**
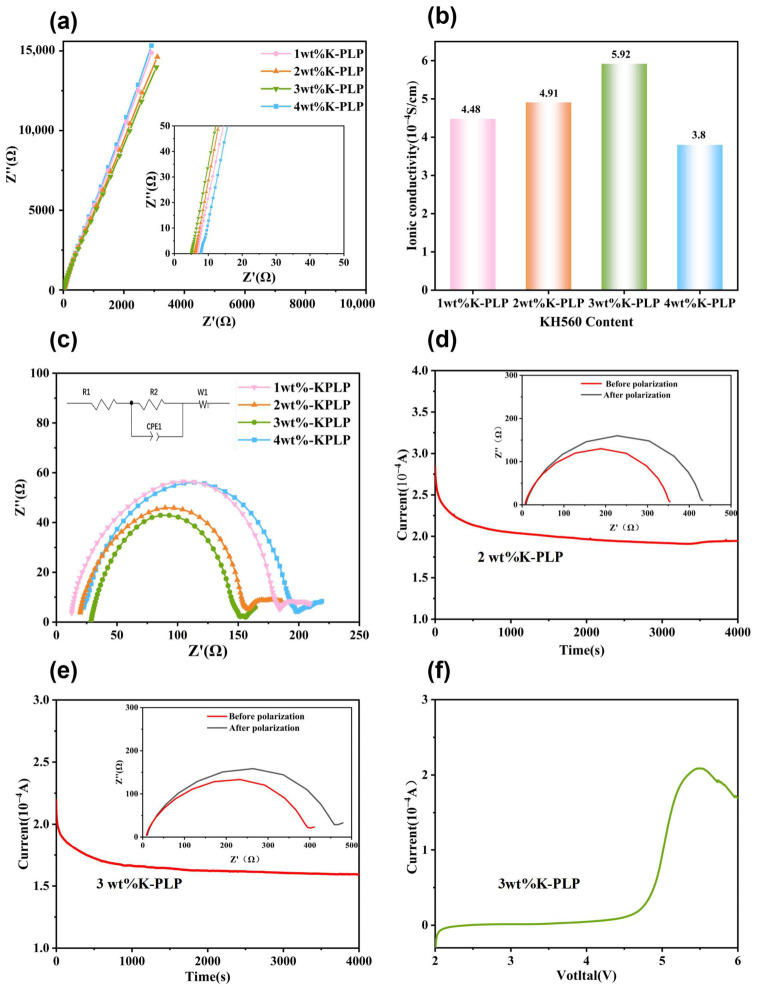
(**a**) EIS plots of 1 wt%~4 wt% K-PLP. (**b**) Ionic conductivity of 1 wt%~4 wt% K-PLP. (**c**) Interfacial impedance of 1 wt%~4 wt% K-PLP. (**d**) Li^+^ transference number of 2 wt% K-PLP. (**e**) Li^+^ transference number of 3 wt% K-PLP. (**f**) Electrochemical stability window of 3 wt% K-PLP.

Ionic conductivity is a core indicator determining the electrochemical performance of composite electrolytes. In this study, the impedance behaviors of composite electrolytes with different KH560 grafting contents (1 wt%, 2 wt%, 3 wt%, 4 wt%) were tested by Electrochemical Impedance Spectroscopy (EIS), and their room-temperature ionic conductivities were calculated. The results are shown in Figure 4a,c. The variation law of impedance spectra that when the KH560 grafting content increases from 1 wt% to 3 wt%, both the bulk impedance and interfacial impedance of the electrolytes decrease continuously; while when the grafting content rises to 4 wt%, the diameter of the high-frequency semicircle increases again and the impedance rises significantly. The fitted data is shown in Table 1. Figure 4b presents the ionic conductivity results: the room-temperature ionic conductivity of 1 wt% K-PLP is 4.48 × 10^−4^ S cm^−1^, that of 2 wt% K-PLP increases to 4.91 × 10^−4^ S cm^−1^, 3 wt% K-PLP reaches the optimal value of 5.92 × 10^−4^ S cm^−1^, while the ionic conductivity of 4 wt% K-PLP drops to 3.80 × 10^−4^ S cm^−1^, which is even lower than that of 1 wt% K-PLP. The variation law of ionic conductivity can be explained by the competitive mechanism of “ion transport channels” and “agglomeration effect”: LLTO in KH560-PDA@LLTO itself possesses a high lithium ion transference number, and uniformly dispersed inorganic fillers can construct continuous “inorganic ion transport channels” in the polymer matrix. Meanwhile, the modification effect of KH560 improves the interfacial compatibility between fillers and the matrix and reduces the interfacial impedance. However, excessive grafting content not only destroys the continuity of “inorganic ion transport channels”, but also increases the transport resistance of lithium ions. In addition, voids are easily formed between agglomerates and the polymer matrix, leading to an increase in interfacial impedance and thus a significant decline in ionic conductivity. This result confirms that 3 wt% is the optimal grafting content for KH560-PDA@LLTO, which can maximize the ion transport performance of composite electrolytes. Prior to this work, the ionic conductivity of pristine PVDF-HFP electrolyte was measured to be 3.23 × 10^−5^ S cm^−1^. The ionic conductivity of the dual-modified composite electrolyte prepared in this study is improved by one order of magnitude compared with the pristine electrolyte, which reveals that the KH560/PDA co-grafting modification can optimize filler dispersion and reduce ion transport resistance, thus significantly enhancing the ionic conduction performance of PVDF-HFP-based electrolytes at the microscopic structural level.

The potentiostatic polarization curves and impedance spectra in Figure 4d,e show that as the grafting content of KH560 increases from 2 wt% to 3 wt%, the lithium-ion transference number of the composite membrane rises from 0.84 to 0.88. This change reflects the significant regulatory effect of filler modification on the ion transport behavior inside the membrane. During the potentiostatic polarization process, there are obvious differences in the steady-state current between the 2 wt% and 3 wt% composite membranes: the 3 wt% composite membrane has a higher steady-state current, and the change in interfacial impedance before and after polarization is more gentle, indicating that the high-content modified filler can more effectively construct continuous lithium-ion transport channels and inhibit anion migration. The high lithium-ion transference number originates from three synergistic effects. The polydopamine coating on LLTO is rich in polar hydroxyl and amino groups, which can anchor lithium salt anions via hydrogen bonding and electrostatic interaction to greatly restrain anion migration and reduce the conductive contribution of anions. In addition, LLTO particles possess intrinsic exclusive transport tunnels for Li^+^, which only enable efficient lithium-ion conduction while barely transferring anions or electrons, further decreasing the proportion of mixed conductance. Meanwhile, the KH560 silane coupling agent covalently links fillers with the PVDF-HFP matrix to achieve uniform filler dispersion, preventing the formation of electronic conductive pathways induced by particle agglomeration and eliminating interference from electronic conduction.

The electrochemical stability window determines the cathode material systems compatible with composite electrolytes, and a wide electrochemical window is a prerequisite for developing high-energy-density solid-state lithium batteries [34]. In this study, the electrochemical stability window of the modified composite electrolyte was tested by linear sweep voltammetry (LSV), and the results are shown in Figure 4f. In the scanning voltage range of 2~4.88 V, the current density of the composite electrolyte remains at an extremely low level of the order of 10^−4^ A, with no obvious increase in oxidation current, indicating that the electrolyte does not undergo electrochemical behaviors such as oxidative decomposition and side reactions within this voltage range and possesses excellent electrochemical stability. When the scanning voltage exceeds 4.88 V, the current density rises sharply and the upward trend continues, corresponding to the irreversible oxidative decomposition reaction of the composite electrolyte. Therefore, the electrochemical stability window of this modified composite electrolyte is determined to be 4.88 V. The introduction of KH560-PDA@LLTO inorganic fillers forms a stable interfacial phase between the polymer matrix and electrodes, inhibiting the oxidative decomposition of the electrolyte under high voltage. Meanwhile, the silanization modification of KH560 enhances the molecular bonding energy of the electrolyte and raises its oxidative decomposition potential [35]. The wide electrochemical stability window of 4.88 V is compatible with various cathode materials.

**Figure 5:**
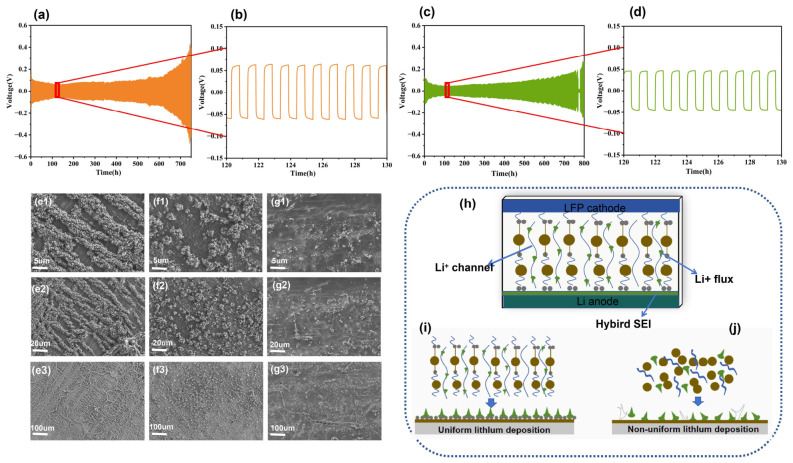
(**a**,**b**) Lithium interfacial stability of 2 wt% K-PLP. (**c**,**d**) Lithium interfacial stability of 3 wt% K-PLP. (**e1**–**e3**) SEM images of Li foil cycled with LP. (**f1**–**f3**) SEM images of Li foil cycled with PLP. (**g1**–**g3**) SEM images of Li foil cycled with 3 wt% K-PLP. (**h**–**j**) Schematic illustrations of Li deposition behavior with and without interface regulation.

The interfacial stability between the lithium-metal anode and electrolyte is a key factor determining the long-cycle performance of solid-state lithium batteries [36]. Unstable interfaces are prone to inducing problems such as lithium dendrite growth and interfacial side reactions, leading to the degradation of battery performance. In this study, Li/electrolyte/Li symmetric cells were assembled, and the long-term lithium stripping/plating cycle performance of K-PLP composite electrolytes was tested at a current density of 0.1 mA/cm^2^. During the continuous cycling for 700 h, the voltage-time curves of the two electrolytes showed significant differences: the voltage hysteresis of the 2 wt% K-PLP electrolyte increased continuously with the extension of cycling time; the voltage polarization in the initial cycle was about ±55 mV, and the polarization voltage increased after 700 h of cycling, as shown in Figure 5a,b. In contrast, the voltage hysteresis of the 3 wt% K-PLP electrolyte was always maintained at a low level of about ±50 mV, the voltage platform was stable during the 750 h cycling, and there was no significant increase in polarization, as shown in Figure 5c,d. The results of partial enlarged views further highlight the interfacial advantages of 3 wt% K-PLP. This excellent interfacial stability stems from two aspects: first, the good mechanical properties of K-PLP composite electrolytes can inhibit the nucleation and growth of lithium dendrites through mechanical blocking; second, the modification effect of KH560 enables the electrolyte to form a stable Li^+^-rich interfacial phase with the lithium-metal anode, reducing the occurrence of interfacial side reactions. Meanwhile, the uniform ion transport channels can realize the uniform deposition of lithium ions and avoid the formation of dendrites due to local lithium-metal enrichment [37].

The SEM images clearly demonstrate significant differences in protective efficacy against lithium-metal anodes among three composite solid electrolytes (LLTO/PVDF-HFP, PDA@LLTO/PVDF-HFP, and KH560PA@LLTO/PVDF-HFP) after 100 cycles. Unmodified lithium plates Figure 5(e1–e3) exhibited extensive, heterogeneous dendritic deposits and pronounced grooved structures on their surfaces, indicating uncontrolled lithium ion deposition during cycling that resulted in abundant lithium dendrites and “dead lithium” formation. This severe surface degradation not only compromises battery cycle stability but also poses serious safety risks. In PDA-modified PLP systems Figure 5(f1–f3), although aggregates remained present, their distribution became significantly reduced and more uniform compared to unmodified systems, with large-scale dendritic structures effectively suppressed. This improvement stems from PDA’s enhancement of filler–polymer matrix interfacial compatibility and optimized interfacial lithium ion transport kinetics, facilitating more homogeneous deposition. The K-PLP system Figure 5(g1–g3) with dual KH560PDA modification demonstrated optimal protection performance, maintaining a relatively flat and dense surface morphology across all magnification levels with negligible dendrite formation and large-scale deposits. This achievement is attributed to the dual regulatory effects of the KH560-PDA-modified layer on LLTO filler: On one hand, the silane coupling agent KH560 effectively improves the interfacial wettability and adhesion between inorganic filler and polymer matrix, reducing interfacial impedance. On the other hand, the adhesive properties of PDA further stabilize the interface, induce uniform lithium-ion deposition, and effectively suppress lithium dendrite growth, thereby significantly enhancing battery cycle stability and safety. Additionally, KH560 reacts with lithium sheets to form a dense solid electrolyte interface (SEI) film, further inhibiting lithium dendrite penetration [22]. Figure 5h further illustrates the mechanism of this composite electrolyte in LFP all-solid-state batteries, where LLTO particles form a continuous ion transport network that guides Li^+^ ions from the lithium anode to the LFP cathode with high efficiency and directionality, ensuring uniform lithium deposition. Figure 5i,j visually compare lithium deposition behaviors with and without interface regulation. Under the regulation of KH560-PDA@LLTO composite electrolyte, a stable hybrid solid electrolyte interface forms, promoting uniform and planar lithium-metal deposition. In contrast, ineffective interface regulation results in disordered, protruding non-uniform deposition prone to lithium dendrite growth. Overall, the core function of KH560-PDA coating LLTO lies in constructing a stable hybrid interface layer and regulating Li^+^ transport pathways, thereby achieving uniform lithium deposition and providing critical structural and interface control strategies for improving all-solid-state lithium batteries’ cycle stability and safety.

**Figure 6:**
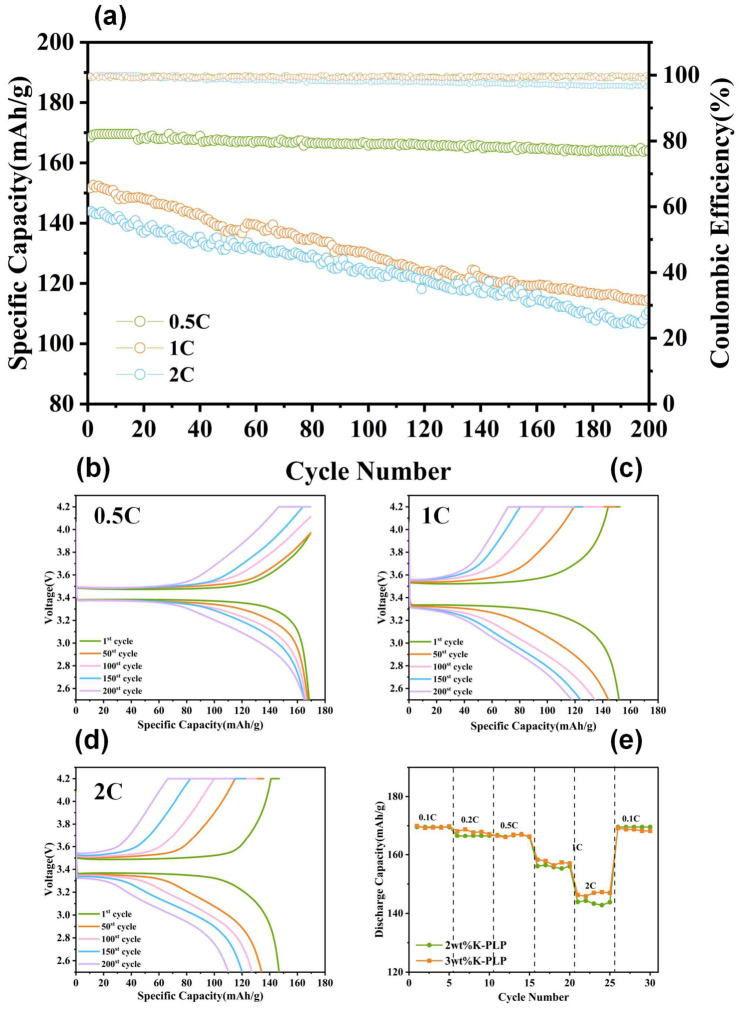
(**a**) Long-cycle performance of 3 wt% K-PLP at 0.5 C, 1 C, and 2 C. (**b**–**d**) Charge–discharge curves at 0.5 C, 1 C, and 2 C. (**e**) Rate performance of 2 wt% K-PLP and 3 wt% K-PLP.

To evaluate the practical application performance of composite electrolytes, Li/composite electrolyte/LFP full cells were assembled in this study, and their electrochemical performance under different cycles was systematically tested. The results are shown in Figure 6a, which presents the cycling performance curves of 3 wt% K-PLP at rates of 0.5 C, 1 C and 2 C. At the three rates of 0.5 C, 1 C and 2 C, the specific capacity of the cells gradually decays with the increase in cycling numbers. However, the cell delivers the highest initial specific capacity of 168.52 mAh/g at 0.5 C, and maintains a relatively high specific capacity after 200 cycles with a capacity retention rate of 97.3%. When the rate increases to 1 C and 2 C, both the initial specific capacity and cycling stability of the cells decline, which is attributed to the limited lithium-ion diffusion kinetics at high rates. Even under cycling at a high rate of 2 C, the discharge specific capacity still reaches 151.59 mAh/g, and a capacity retention of 75.7% is maintained after 200 cycles. The charge–discharge curves in Figure 6b–d further indicate that the voltage difference between the charge and discharge platforms gradually increases and the polarization phenomenon intensifies as the cycling number rises, which is related to the continuous loss of active materials and the increase in interfacial impedance. The above results demonstrate that the as-prepared composite electrolyte possesses excellent electrochemical performance at medium and low rates, and can meet the basic application requirements of solid-state lithium batteries.

**Figure 7:**
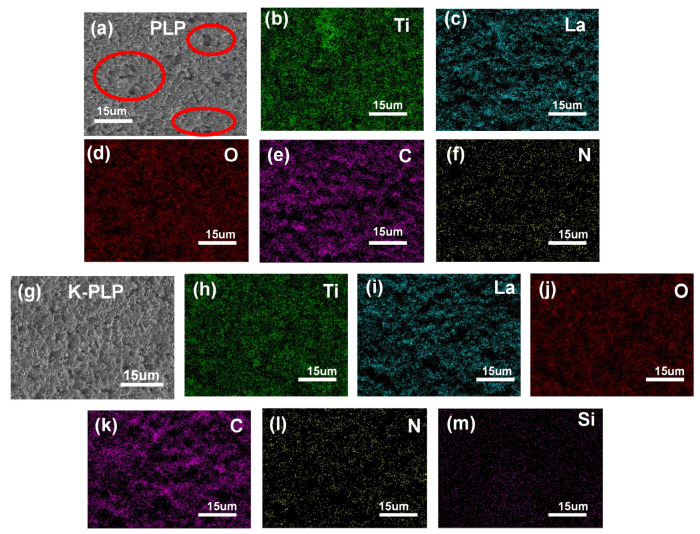
(**a**) Surface SEM image of PLP. (**b**–**f**) EDS elemental mappings of PLP. (**g**) Surface SEM image of K-PLP. (**h**–**m**) EDS elemental mappings of K-PLP.

The cycled composite membranes were subjected to SEM testing. Figure 7 shows the SEM-EDS elemental mapping distribution of PLP and K-PLP composite electrolytes. The SEM-EDS mapping results of PLP are presented in Figure 7b–f in which elements including C, N, O, Ti and La are detected. In contrast, as shown in Figure 7h–m, elements such as C, N, O, Ti, La and Si are uniformly distributed without obvious agglomeration in the K-PLP sample. The uniform distribution of Si elements directly proves that KH560-PDA@LLTO is well dispersed in the polymer matrix. The above results indicate that the KH560-PDA@LLTO inorganic filler is evenly dispersed in the composite electrolyte with favorable interfacial compatibility, laying a structural foundation for improving the comprehensive performance of the electrolyte. The SEM images in Figure 7a,g show that pores exist in the cycled PLP, as highlighted by the red circles, while the pores in K-PLP are significantly reduced. According to the SEM analysis of lithium-metal stability, the addition of KH560 can inhibit the growth of lithium dendrites. Therefore, the reduction of tiny pores in K-PLP is attributed to the fact that the introduction of KH560 suppresses lithium dendrite growth and thus reduces the penetration of lithium dendrites.

Figure 6e shows the discharge capacity curves of full cells assembled with 2 wt% K-PLP and 3 wt% K-PLP composite electrolytes at different rates. At an initial rate of 0.1 C, the discharge specific capacities of both cells reach approximately 169 mAh/g, close to the theoretical specific capacity of LFP (170 mAh/g), indicating excellent interfacial compatibility between the composite electrolytes and the LFP cathode. When the rate is sequentially increased to 0.2 C, 0.5 C, 1 C and 2 C, the discharge capacities of the cells gradually decrease, while the capacities of the 3 wt% K-PLP cell are higher than those of the 2 wt% K-PLP cell at each rate. When the rate recovers from 2 C back to 0.1 C, the discharge capacities of both cells can quickly return to the initial level with a capacity recovery rate of over 99.8%, demonstrating good reversibility of the cell structure and electrolyte/electrode interface without irreversible damage. The core reason why 3 wt% K-PLP exhibits superior rate performance compared with 2 wt% K-PLP lies in its higher ionic conductivity and lower interfacial impedance, which enable more efficient lithium-ion transport at high current densities and meet the requirements for rapid electrode reactions. In summary, the Li/LFP full cell assembled with the 3 wt% K-PLP composite electrolyte possesses both outstanding cycling stability and rate capability, fully verifying the practical application value of this composite electrolyte.

To elucidate the synergistic advantages of KH560/PDA co-grafted LLTO over singly modified fillers, key electrochemical performances are summarized in Table 2. Comparative data reveal that single PDA coating only relies on non-covalent interactions and suffers from poor long-cycle stability; fillers modified solely with KH560 possess weak interfacial bonding with polymers and only deliver high ionic conductivity at elevated temperatures. The synergistic dual modification simultaneously optimizes organic–inorganic interfacial compatibility, lithium-ion transport kinetics and lithium-metal anode compatibility, endowing the composite electrolyte in this work with optimal comprehensive electrochemical performance at room temperature.

## 4. Conclusions

This work develops a high-performance PVDF-HFP-based composite solid electrolyte modified by KH560-PDA synergistically treated LLTO. The dual modification constructs a strong covalent interface between LLTO and the polymer matrix, greatly improving dispersion, mechanical properties, and electrochemical performance. The 3 wt% KH560 grafting ratio is optimal, yielding: room-temperature ionic conductivity: 5.92 × 10^−4^ S cm^−1^; Li^+^ transference number: 0.88; electrochemical stability window: 4.88 V; stable Li stripping/plating for >700 h at ±50 mV polarization; and Li/LFP full cell: 168.52 mAh/g at 0.5 C with 97.3% retention over 200 cycles. The modified electrolyte effectively suppresses lithium dendrite growth and enhances interfacial stability. Compared with single KH560 or PDA modification, the KH560/PDA co-grafting strategy achieves synergistic effects: covalent crosslinking stabilizes the interfacial structure, while polar groups optimize filler dispersion and build Li+ transport pathways, thereby improving the comprehensive performance of the composite electrolyte. This study provides a reliable interfacial engineering strategy for designing advanced composite electrolytes toward practical, safe, and long-life solid-state lithium-metal batteries.

## Figures and Tables

**Table 1:** Interface impedance fitting parameters for 1 wt%–4 wt% concentrations.

Sample	Rs/Ω (95%CI)	Rct/Ω (95%CI)	CPE-P	Warburg Coefficient/Ω·s−1/2
1 wt% K-PLP	18.5 ± 0.8	173 ± 3	0.87	4.08
2 wt% K-PLP	16.9 ± 0.7	130 ± 3	0.88	4.72
3 wt% K-PLP	14.2 ± 0.6	124 ± 2	0.90	5.41
4 wt% K-PLP	17.6 ± 0.7	175 ± 3	0.86	3.95

**Table 2:** Electrochemical performance comparison of composite solid electrolytes with various modified inorganic fillers.

Modification Method	Modified Filler	Magnitude of Room-Temperature Ionic Conductivity of Electrolytes	Lithium Transference Number (Li^+^)	Full-Battery Capacity Retention	Source of Literature
Single PDA coating	LLZTO	1.11 × 10^−4^ S cm^−1^	0.40	83%@100 cycles (0.1 C)	Huang et al. [38]
Single PDA coating	LLTO	4.16 × 10^−4^ S cm^−1^	0.45	78.8%@400 cycles (0.4 C)	Jia et al. [13]
Single modification with KH560	@InF3	2.1 × 10^−4^ S cm^−1^	0.73	83.6%@800 cycles (10 C)	Chen et al. [37]
Single modification with KH560	Porous LLTO	2.37 × 10^−3^ S cm^−1^ (60 °C)	0.57	-	CHI et al. [19]
Co-grafting Modification with KH560/PDA	LLTO	5.92 × 10^−4^ S cm^−1^	0.88	97.3%@200 cycles (0.5C) 75.7%@200 cycles (2 C)	This work

## Data Availability

The original contributions presented in this study are included in the article. Further inquiries can be directed to the corresponding author.
